# Routine Screening for Antibodies to Human Immunodeficiency Virus in the U.S. Armed Forces, Active and Reserve Components, January 2019–June 2024

**Published:** 2024-10-20

**Authors:** Armed Forces Health Surveillance Division Defense Health Agency, US

## Abstract

**What are the new findings?:**

From January 2023 through June 2024, approximately 1.8 million service members (active component, Guard, and reserve) were tested for antibodies to HIV, and 403 (0.22 per 1,000 tested) were identified as HIV-antibody positive. Of the 403 new HIV infections that were identified during this period, only 10 (2.5%) were among female service members.

**What is the impact on readiness and force health protection?:**

The HIV-antibody seropositivity rates first reported in *MSMR* 3 decades ago remain comparable to rates presented in 2023, under scoring a continued value of HIV testing programs. The cost-effectiveness of HIV testing strategies, differentiated by universal or indications-based testing following military accession, may be instructive to further understand the value of current screening efforts in different clinical settings.

## 1 BACKGROUND

The U.S. Department of Defense (DOD) has conducted an active surveillance program for HIV since 1986, when reliable screening methods became widely available. This program consists of screening all service members at specific points in time: prior to entry (all accessions must be HIV-negative prior to the start of service), before deployment or any change in status (e.g., change in component, between branches, or commissioning), and once every 2 years while a member of the U.S. military.^1^

While infection with HIV currently disqualifies applicants for entry into U.S. military service, this policy may be affected by a recent federal court ruling that the DOD cannot ban HIV-positive people with undetectable viral loads from joining the military.^2^ Due to significant advances in the diagnosis, treatment, and prevention of HIV, in June 2022 the DOD amended policies to prevent HIV-positive service members with an undetectable viral load from being discharged or separated solely on the basis of their HIV status.^1^ In addition, HIV-positive personnel are not non-deployable solely for a positive status; decisions related to deployability should be made on a case-by-case basis and must be justified by a service member’s inability to perform the duties to which he or she would be assigned.^3^

Summaries of HIV seropositivity for members of the U.S. military have been published with *MSMR* since 1995. The current report summarizes numbers and trends of newly identified HIV-antibody seropositivity from January 1, 2019 through June 30, 2024 among military members of 5 services under the active and reserve components of the U.S. Armed Forces, in addition to the Army and Air Force National Guard.

## 2 METHODS

The surveillance population included all individuals eligible for HIV antibody screening from January 1, 2019 through June 30, 2024 while serving in the active or reserve components of the U.S. Army, Navy, Air Force, Marine Corps, or Coast Guard. Space Force service members were categorized as Air Force for this analysis. All individuals who were tested, and all initial detections of antibodies to HIV, through U.S. military medical testing programs were ascertained from the Department of Defense Serum Repository (DODSR) specimens accessioned to the Defense Medical Surveillance System (DMSS). Due to changes in data processing, positive specimens for Navy and Marine Corps service members are no longer accessioned in DODSR and DMSS. To account for this limitation, data for the Navy and Marine Corps were obtained from the Navy Bloodborne Infection Management Center (NBMIC); the total number of HIV infections from 2022 through June 30, 2024 were ascertained from the Navy’s HIV Management System and NBMIC end-of-year reports.

An incident case of HIV-antibody seropositivity was defined as an individual with positive HIV test results on 2 different, serial specimens. Individuals who had just 1 positive result without a subsequent negative result were also defined as positive, to capture those who had yet to test positive for a second time.

Non-positive HIV samples from Navy service members remain documented in DODSR and accessioned through DMSS; thus, the total number of HIV-positive tests were acquired from DMSS to calculate seropositivity rates as a standardized methodology for all services. Annual rates of HIV seropositivity among service members were calculated by dividing the number of incident cases of HIV antibody seropositivity during each calendar year by the number of individuals who were tested at least once during the relevant calendar year. Rates were further stratified by service, component, and sex.

## 3 RESULTS

From January 2023 through June 2024, approximately 1.8 million U.S. service members (of the active component, Guard, and reserve) were tested for antibodies to HIV, and of those individuals tested, 403 (0.22 per 1,000) were identified as HIV-antibody positive. Of the 403 new HIV infections identified during this period, only 10 (2.5%) occurred in female service members.

**U.S. Army**

**Figure 1:**
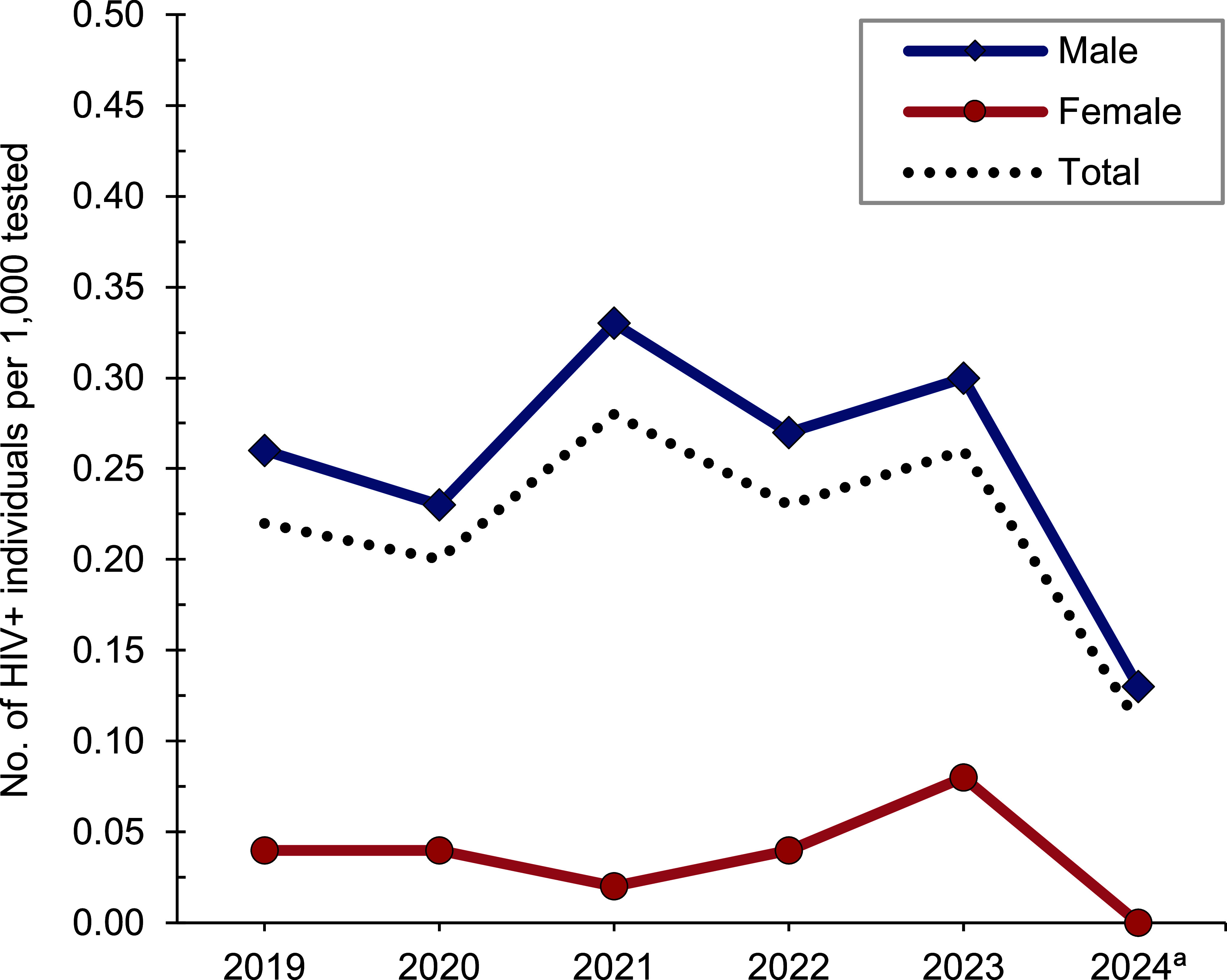
HIV Antibody Seropositivity Rates by Sex, Active Component, U.S. Army, January 2019–June 2024

*Active component*: From January 2023 through June 2024, a total of 458,315 U.S. Army active component soldiers were tested for HIV antibodies, and 97 were identified as HIV-antibody positive (seropositivity: 0.21 per 1,000 tested) (**Table 1**). During the surveillance period, annual seropositivity rates fluctuated between a low of 0.11 per 1,000 tested in 2024 (through June) and a high of 0.28 per 1,000 tested in 2021 (**Table 1**, **Figure 1**). Annual seropositivity rates for male active component soldiers were considerably higher than those of female active component soldiers (**Figure 1**). In 2023, 1 new HIV infection on average was detected among active component soldiers per 4,682 screening tests (**Table 1**). Of the 401 active component soldiers diagnosed with HIV infections since 2019, a total of 250 (62.3%) were still in military service in 2024.

*Army National Guard*: From January 2023 through June 2024, a total of 283,865 U.S. Army National Guard members were tested for HIV antibodies, and 84 soldiers were identified as HIV-antibody positive (seropositivity: 0.30 per 1,000 tested) (**Table 2**). On average, 1 new HIV infection was detected in 2023 among Army National Guard soldiers per 4,466 screening tests. Of the 294 National Guard soldiers who tested positive for HIV since 2019, a total of 201 (68.4%) were still in service in 2024.

*Army Reserve*: From January 2023 through June 2024, a total of 111,713 U.S. Army Reserve members were tested for HIV antibodies, and 39 were identified as HIV-antibody positive (seropositivity: 0.35 per 1,000 tested) (**Table 3**). During 2023, on average 1 new HIV infection was detected among Army reservists per 3,308 screening tests. Of the 168 Army reservists diagnosed with HIV infections since 2019, a total of 107 (63.7%) were still in service in 2024.

**U.S. Navy**

**Figure 2:**
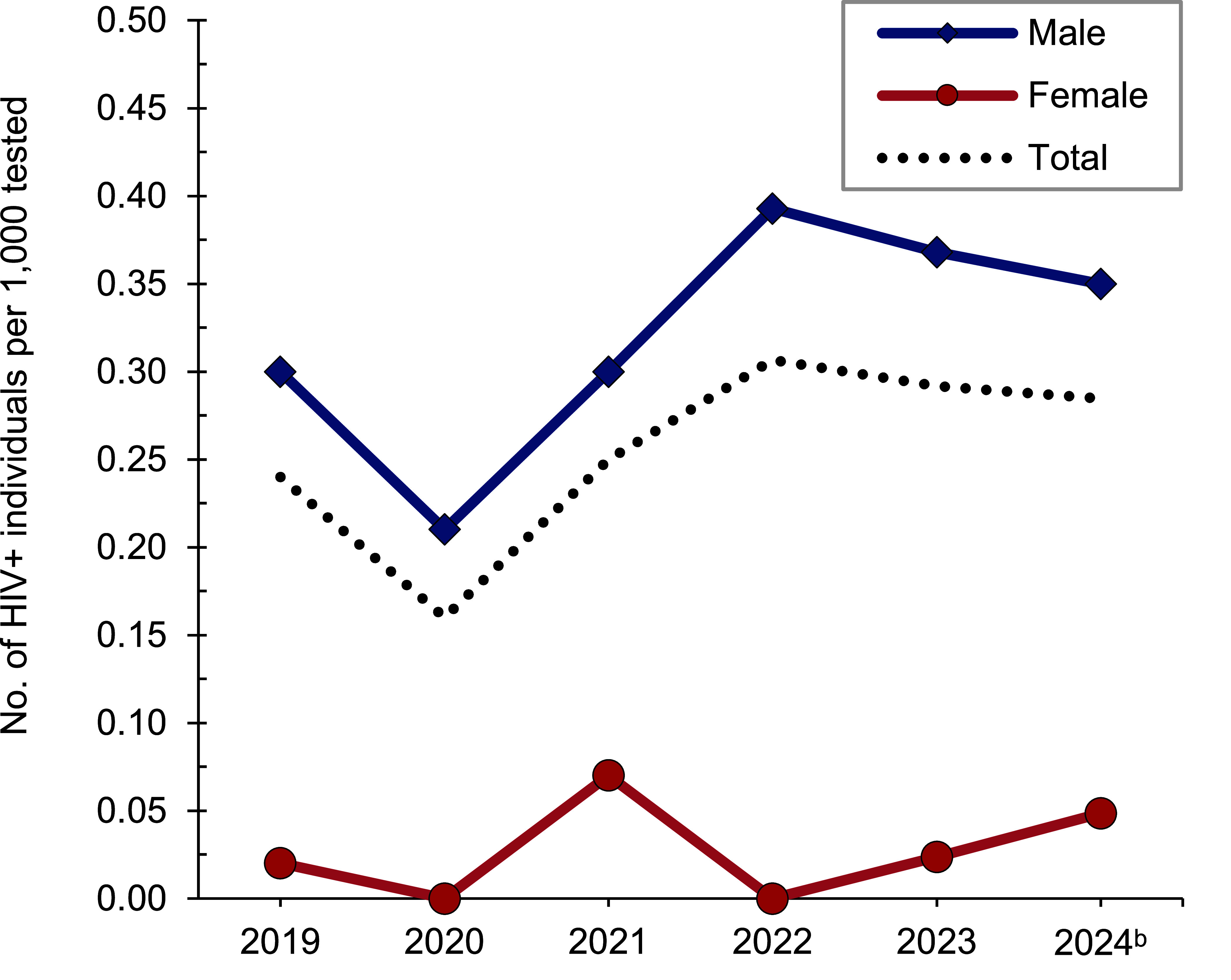
HIV Antibody Seropositivity Rates^a^ by Sex, Active Component, U.S. Navy, January 2019–June 2024

*Active component*: A total of 286,804 members of the U.S. Navy active component were tested for HIV antibodies from January 2023 through June 2024, and 83 sailors were identified as HIV-antibody positive (seropositivity: 0.29 per 1,000 tested) (**Table 4**). During the surveillance period, annual seropositivity rates fluctuated between a low of 0.16 per 1,000 tested in 2020 and a high of 0.31 per 1,000 tested in 2022 (**Table 4**, **Figure 2**). Annual seropositivity rates for male active component sailors were considerably higher than those of female active component soldiers (**Figure 2**). During 2023, on average, 1 new HIV infection was detected among active component sailors per 3,983 screening tests. Of the 283 active component sailors diagnosed with HIV infections since 2019, a total of 208 (73.5%) were still in service in 2024.

*Navy Reserve*: From January 2023 through June 2024, a total of 44,375 members of the U.S. Navy Reserve were tested for HIV antibodies, and 6 sailors were identified as HIV-antibody positive (seropositivity: 0.14 per 1,000 tested) (**Table 5**). On average, 1 new HIV infection was detected in 2023 among Navy reservists per 10,879 screening tests. Of the 38 reserve component sailors diagnosed with HIV infections since 2019, a total of 25 (65.8%) were still in military service in 2024.

**U.S. Air Force**

**Figure 3:**
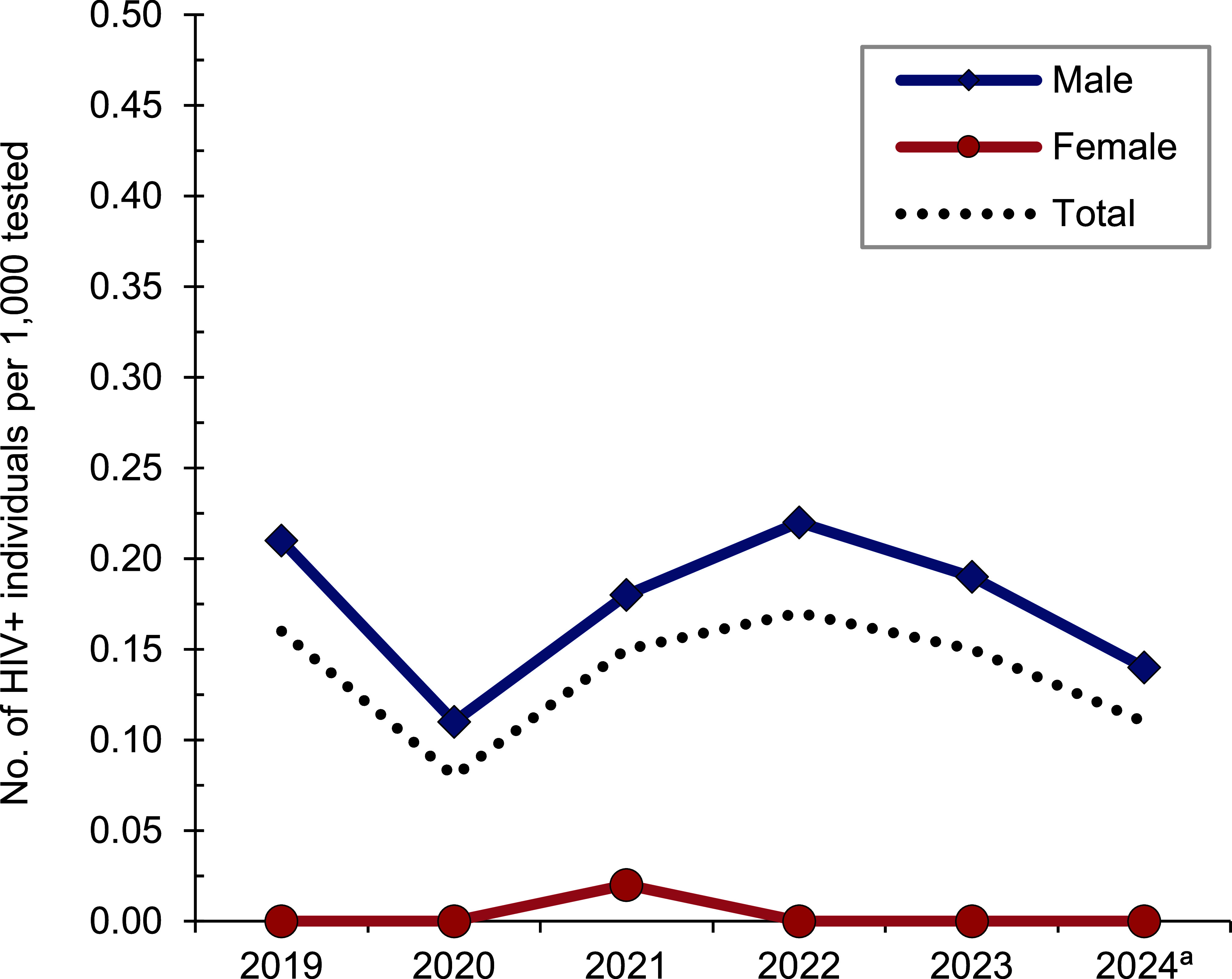
HIV Antibody Seropositivity Rates by Sex, Active Component, U.S. Air Force, January 2019–June 2024

*Active component*: From January 2023 through June 2024, a total of 282,636 active component members of the U.S. Air Force were tested for HIV antibodies, and 39 Air Force members were diagnosed with HIV infection (seropositivity: 0.14 per 1,000 tested) (**Table 6**). On average, 1 new HIV infection was detected in 2023 among active component Air Force members per 8,595 screening tests. Of the 152 active component Air Force members diagnosed with HIV infections since 2019, 101 (66.4%) were still in military service in 2024. During the surveillance period, seropositivity rates among male members ranged from a low of 0.11 per 1,000 tested in 2020 to a high of 0.22 per 1,000 tested in 2022 (**Figure 3**).

*Air National Guard*: From January 2023 through June 2024, a total of 84,470 members of the Air National Guard were tested for HIV antibodies, and 8 Air National Guard members were diagnosed with HIV infection (seropositivity: 0.09 per 1,000 airmen tested) (**Table 7**). During 2023, on average 1 new HIV infection was detected among Air National Guard members per 14,137 screening tests. Of the 32 Air National Guard members diagnosed with HIV infections since 2019, 25 (78.1%) were still in service in 2024.

*Air Force Reserve*: From January 2023 through June 2024, a total of 49,078 members of the Air Force Reserve were tested for HIV antibodies, and 6 Air Force reservists were diagnosed with HIV infections (seropositivity: 0.12 per 1,000 tested) (**Table 8**). On average, in 2023 1 new HIV infection was detected among Air Force reservists per 9,725 screening tests. Of the 38 reservists in the Air Force diagnosed with HIV infections since 2019, 28 (73.7%) were still in military service in 2024.

**U.S. Marine Corps**

**Figure 4:**
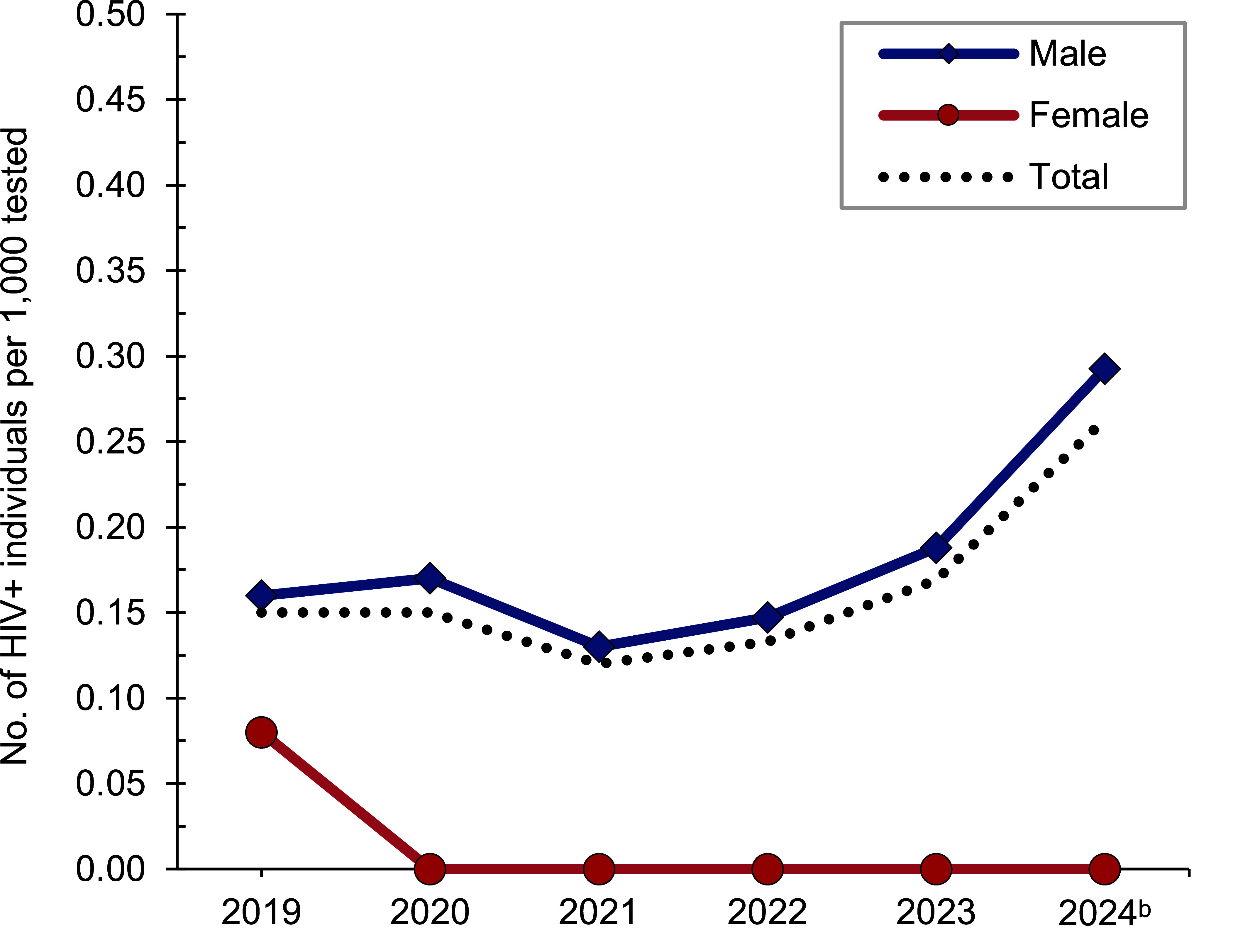
HIV Antibody Seropositivity Rates^a^ by Sex, Active Component, U.S. Marine Corps, January 2019–June 2024

*Active component*: From January 2023 through June 2024, a total of 161,928 U.S. Marine Corps active component members were tested for HIV antibodies, and 32 were identified as HIV-antibody positive (seropositivity: 0.20 per 1,000 tested) (**Table 9**). Annual seropositivity rates rose from a low of 0.12 per 1,000 tested in 2021 and a high of 0.26 per 1,000 tested at mid-year 2024 (**Table 9**, **Figure 4**). In 2023, on average, 1 new HIV infection per 6,820 screening tests was detected among active component marines. Of the 102 active component marines diagnosed with HIV infections since 2019, a total of 54 (52.9%) were still in service in 2024.

*Marine Corps Reserve*: From January 2023 through June 2024, a total of 29,271 Marine Corps Reserve members were tested for antibodies to HIV, and 8 reservists were identified as HIV-antibody positive (seropositivity: 0.27 per 1,000 tested) (**Table 10**). During 2023, on average, 1 new HIV infection was detected among Marine Corps reservists per 7,192 screening tests. All 8 reservists diagnosed with HIV infection since 2023 were still in military service in 2024.

**U.S. Coast Guard**

*Active component*: From January 2023 through June 2024, a total of 27,709 U.S. Coast Guard active component members were tested for antibodies to HIV, and 1 was identified as HIV-antibody positive (**Table 11**). Of the 5 active component Coast Guardsmen diagnosed with HIV infections since 2019, a total of 3 (60.0%) were still in service in 2024.

*Coast Guard Reserve*: From January 2023 through June 2024, a total of 4,448 U.S. Coast Guard Reserve members were tested for HIV antibodies, with none identified as HIV-antibody positive (**Table 12**).

## 4 DISCUSSION

The U.S. military has conducted routine screening for antibodies to HIV among all civilian applicants for service and all service members for more than 30 years.^4,5,6,7^ In 1995 the U.S. Army tested approximately 1.1 million specimens a year, demonstrating an economically efficient, large-scale model for HIV testing.^8^ The first *MSMR* article to publish results from HIV screening programs indicates that antibody seropositivity rates in 1994 for the Army active duty (0.19 per 1,000 soldiers) and reserve component (0.23 per 1,000 soldiers) remain comparable to rates presented in 2023.^9^ Three decades later, this comparison underscores a continued value of HIV testing programs. The cost-effectiveness of HIV testing strategies, delineated by universal or indications-based testing after entry into the military, may be instructive to understand the value of current screening efforts in different clinical settings.

Archived surveillance data also reflect improved retention of HIV-positive service members, in alignment with recent DOD policy that recognizes significant advances in the diagnosis, prevention, and treatment of the disease. From 1990 to 1994, a total of 889 active and reserve component soldiers were diagnosed with HIV-1 infection. By 1995, only 234 (26.0%) were still in service.^9^ Today, a comparative retention figure for active component Army service members has increased to 62.3% (250 of 401 soldiers diagnosed since 2019 are still in service as of 2024). Retention of HIV-positive service members differs by component and service branch, with highest retention demonstrated for the Air Force National Guard (78.1%), Air Force Reserve (73.7%), and active component Navy (73.5%); however, these figures are not adjusted for overall retention differences across the force.

The most recent active component Army results indicate a substantial decline of new HIV infections as of June 2024, dropping from 0.26 per 1,000 soldiers in 2023 to 0.11 per 1,000 soldiers as of mid-year 2024. An inverse trend was observed for the active component Marines, doubling between 2021 and mid-year 2024 (from 0.12 per 1,000 marines to 0.26 per 1,000 marines). For both services, the mid-year 2024 HIV seropositivity rates were higher for the Army Reserve/National Guard and Marine Corps Reserves in comparison to the respective active component.

Routine screening of all civilian applicants for service and routine periodic testing of all active and reserve component members of the services have been fundamental components of the military’s HIV control and clinical management efforts.^10^ Previous *MSMR* reports presented HIV screening results for civilian applicants to the military service; however, these data are no longer available in the Defense Medical Surveillance System (DMSS), as the U.S. Military Entrance Processing Command stopped reporting data to the DMSS at the end of calendar year 2020. Thus, the data presented in this report reflect service members who had a negative HIV test upon entry to military service, followed by a positive test during uniformed service.

The results presented in this report should not be generalized to the U.S. population. Data from HIV screening in U.S. military populations are based on a negative test prior to entry, as well as voluntary service. In countries with universal conscription, compulsory testing in samples of military recruits will be more representative of the young adult population.^10^ Following pre-accession screening of military recruits, routine screening represents relatively recently acquired HIV infections (i.e., infections acquired since the most recent negative test of each affected individual).

## Figures and Tables

**Table 1:** New Diagnoses of HIV Infections, by Sex, Active Component, U.S. Army, January 2019–June 2024

Year	Total HIV Tests	Total Persons Tested	Males Tested	Females Tested	Total New HIV(+)	New HIV(+) Males	New HIV(+) Females	Overall Rate per 1,000 Tested	Male Rate per 1,000 Tested	Female Rate per 1,000 Tested	HIV(+) Still in Military Service in 2024
2019	439,605	345,647	289,716	55,931	77	75	2	0.22	0.26	0.04	33
2020	398,309	322,329	269,966	52,363	65	63	2	0.20	0.23	0.04	31
2021	403,656	323,460	270,836	52,624	90	89	1	0.28	0.33	0.02	48
2022	373,978	306,685	256,698	49,987	72	70	2	0.23	0.27	0.04	52
2023	374,535	303,642	254,111	49,531	80	76	4	0.26	0.30	0.08	69
2024^a^	171,329	154,673	128,736	25,937	17	17	0	0.11	0.13	0.00	17
Total	2,161,412	1,756,436	1,470,063	286,373	401	390	11	0.23	0.27	0.04	250

Abbreviation: HIV, human immunodeficiency virus; +, positive.

^a^Through Jun. 30, 2024.

**Table 2:** New Diagnoses of HIV Infections, by Sex, U.S. Army National Guard, January 2019–June 2024

Year	Total HIV Tests	Total Persons Tested	Males Tested	Females Tested	Total New HIV(+)	New HIV(+) Males	New HIV(+) Females	Overall Rate per 1,000 Tested	Male Rate per 1,000 Tested	Female Rate per 1,000 Tested	HIV(+) Still in Military Service in 2024
2019	235,019	202,926	165,307	37,619	60	60	0	0.30	0.36	0.00	24
2020	215,695	189,933	153,399	36,534	61	58	3	0.32	0.38	0.08	34
2021	218,058	190,119	153,998	36,121	50	48	2	0.26	0.31	0.06	32
2022	207,644	179,205	143,897	35,308	39	36	3	0.22	0.25	0.08	32
2023	214,384	186,739	149,526	37,213	48	47	1	0.26	0.31	0.03	43
2024^a^	104,652	97,126	77,253	19,873	36	35	1	0.37	0.45	0.05	36
Total	1,195,452	1,046,048	843,380	202,668	294	284	10	0.28	0.34	0.05	201

Abbreviation: HIV, human immunodeficiency virus; +, positive.

^a^Through Jun. 30, 2024.

**Table 3:** New Diagnoses of HIV Infections, by Sex, U.S. Army Reserve, January 2019–June 2024

Year	Total HIV Tests	Total Persons Tested	Males Tested	Females Tested	Total New HIV(+)	New HIV(+) Males	New HIV(+) Females	Overall Rate per 1,000 Tested	Male Rate per 1,000 Tested	Female Rate per 1,000 Tested	HIV(+) Still in Military Service in 2024
2019	125,864	109,292	81,929	27,363	42	40	2	0.38	0.49	0.07	23
2020	115,381	101,139	75,248	25,891	24	23	1	0.24	0.31	0.04	10
2021	119,109	101,435	75,562	25,873	29	29	0	0.29	0.38	0.00	18
2022	104,385	90,598	67,121	23,477	34	34	0	0.38	0.51	0.00	21
2023	79,399	69,058	50,624	18,434	24	23	1	0.35	0.45	0.05	22
2024^a^	45,786	42,655	31,532	11,123	15	15	0	0.35	0.48	0.00	13
Total	589,924	514,177	382,016	132,161	168	164	4	0.33	0.43	0.03	107

Abbreviation: HIV, human immunodeficiency virus; +, positive.

^a^Through Jun. 30, 2024.

**Table 4:** New Diagnoses of HIV Infections^a^, by Sex, Active Component, U.S. Navy, January 2019–June 2024

Year	Total HIV Tests	Total Persons Tested	Males Tested	Females Tested	Total New HIV(+)	New HIV(+) Males	New HIV(+) Females	Overall Rate per 1,000 Tested	Male Rate per 1,000 Tested	Female Rate per 1,000 Tested	HIV(+) Still in Military Service in 2024
2019	258,370	222,996	176,040	46,956	54	53	1	0.24	0.30	0.02	24
2020	224,610	199,493	156,082	43,411	32	32	0	0.16	0.21	0.00	17
2021	242,437	215,078	168,993	46,085	54	51	3	0.25	0.30	0.07	38
2022	226,492	195,718	152,740	42,978	60	60	0	0.31	0.39	0.00	46
2023	223,050	191,883	149,533	42,350	56	55	1	0.29	0.37	0.02	56
2024^a^	101,731	94,921	74,307	20,614	27	26	1	0.28	0.35	0.05	27
Total	1,276,690	1,120,089	877,695	242,394	283	277	6	0.25	0.32	0.02	208

Abbreviation: HIV, human immunodeficiency virus; +, positive.

^a^Through Jun. 30, 2024.

**Table 5:** New Diagnoses of HIV Infections^a^, by Sex, U.S. Navy Reserve, January 2019–June 2024

Year	Total HIV Tests	Total Persons Tested	Males Tested	Females Tested	Total New HIV(+)	New HIV(+) Males	New HIV(+) Females	Overall Rate per 1,000 Tested	Male Rate per 1,000 Tested	Female Rate per 1,000 Tested	HIV(+) Still in Military Service in 2024
2019	38,721	34,384	26,473	7,911	9	9	0	0.26	0.34	0.00	6
2020	30,251	27,846	21,136	6,710	6	6	0	0.22	0.28	0.00	4
2021	36,498	33,184	25,040	8,144	11	9	2	0.33	0.36	0.25	3
2022	32,233	28,761	21,569	7,192	6	4	2	0.21	0.19	0.28	6
2023	32,638	29,750	22,142	7,608	3	3	0	0.10	0.14	0.00	3
2024^a^	15,588	14,625	10,943	3,682	3	3	0	0.21	0.27	0.00	3
Total	185,929	168,550	127,303	41,247	38	34	4	0.23	0.27	0.10	25

Abbreviation: HIV, human immunodeficiency virus; +, positive.

^a^Through Jun. 30, 2024.

**Table 6:** New Diagnoses of HIV Infections, by Sex, Active Component, U.S. Air Force, January 2019–June 2024

Year	Total HIV Tests	Total Persons Tested	Males Tested	Females Tested	Total New HIV(+)	New HIV(+) Males	New HIV(+) Females	Overall Rate per 1,000 Tested	Male Rate per 1,000 Tested	Female Rate per 1,000 Tested	HIV(+) Still in Military Service in 2024
2019	262,875	209,389	164,445	44,944	34	34	0	0.16	0.21	0.00	18
2020	243,698	194,466	152,311	42,155	16	16	0	0.08	0.11	0.00	10
2021	256,750	208,331	162,312	46,019	31	30	0	0.15	0.18	0.02	18
2022	238,718	190,562	148,669	41,893	32	32	0	0.17	0.22	0.00	22
2023	249,266	191,525	149,674	41,851	29	29	0	0.15	0.19	0.00	23
2024^a^	109,221	91,111	70,986	20,125	10	10	0	0.11	0.14	0.00	10
Total	1,360,528	1,085,384	848,397	236,987	152	151	1	0.14	0.18	0.00	101

Abbreviation: HIV, human immunodeficiency virus; +, positive.

^a^Through Jun. 30, 2024.

**Table 7:** New Diagnoses of HIV Infections, by Sex, U.S. Air National Guard, January 2019–June 2024

Year	Total HIV Tests	Total Persons Tested	Males Tested	Females Tested	Total New HIV(+)	New HIV(+) Males	New HIV(+) Females	Overall Rate per 1,000 Tested	Male Rate per 1,000 Tested	Female Rate per 1,000 Tested	HIV(+) Still in Military Service in 2024
2019	67,333	58,861	46,271	12,590	7	7	0	0.12	0.15	0.00	3
2020	67,947	58,973	46,174	12,799	6	5	1	0.10	0.11	0.08	4
2021	68,111	60,310	47,167	13,143	7	7	0	0.12	0.15	0.00	7
2022	61,356	54,829	42,793	12,036	4	3	1	0.07	0.07	0.08	3
2023	70,685	56,934	44,614	12,320	5	5	0	0.09	0.11	0.00	5
2024^a^	33,964	27,536	21,685	5,851	3	2	1	0.11	0.09	0.17	3
Total	369,396	317,443	248,704	68,739	32	29	3	0.10	0.12	0.04	25

Abbreviation: HIV, human immunodeficiency virus; +, positive.

^a^Through Jun. 30, 2024.

**Table 8:** New Diagnoses of HIV Infections, by Sex, U.S. Air Force Reserve, January 2019–June 2024

Year	Total HIV Tests	Total Persons Tested	Males Tested	Females Tested	Total New HIV(+)	New HIV(+) Males	New HIV(+) Females	Overall Rate per 1,000 Tested	Male Rate per 1,000 Tested	Female Rate per 1,000 Tested	HIV(+) Still in Military Service in 2024
2019	42,210	37,046	26,854	10,192	7	7	0	0.19	0.26	0.01	4
2020	38,943	33,947	24,604	9,343	6	6	0	0.18	0.24	0.00	4
2021	41,589	37,431	27,023	10,408	15	14	1	0.40	0.52	0.10	10
2022	37,273	33,459	24,184	9,275	4	4	0	0.12	0.17	0.00	4
2023	38,901	33,665	24,350	9,315	4	4	0	0.12	0.16	0.00	4
2024^a^	17,984	15,413	10,898	4,515	2	2	0	0.13	0.18	0.00	2
Total	216,900	190,961	137,913	53,048	38	37	1	0.20	0.27	0.02	28

Abbreviation: HIV, human immunodeficiency virus; +, positive.

^a^Through Jun. 30, 2024.

**Table 9:** New Diagnoses of HIV Infections^a^, by Sex, Active Component, U.S. Marine Corps, January 2019–June 2024

Year	Total HIV Tests	Total Persons Tested	Males Tested	Females Tested	Total New HIV(+)	New HIV(+) Males	New HIV(+) Females	Overall Rate per 1,000 Tested	Male Rate per 1,000 Tested	Female Rate per 1,000 Tested	HIV(+) Still in Military Service in 2024
2019	160,052	138,199	125,668	12,531	21	20	1	0.15	0.16	0.08	3
2020	140,662	123,760	112,634	11,126	19	19	0	0.15	0.17	0.00	5
2021	148,035	129,764	117,793	11,971	15	15	0	0.12	0.13	0.00	5
2022	129,466	112,851	101,859	10,992	15	15	0	0.13	0.15	0.00	9
2023	129,578	112,487	101,123	11,364	19	19	0	0.17	0.19	0.00	19
2024^a^	53,195	49,441	44,431	5,101	13	137	0	0.26	0.29	0.00	13
Total	760,988	666,502	603,508	62,994	102	101	1	0.15	0.17	0.02	54

Abbreviation: HIV, human immunodeficiency virus; +, positive.

^a^Through Jun. 30, 2024.

**Table 10:** New Diagnoses of HIV Infections^a^, by Sex, U.S. Marine Corps Reserve, January 2019–June 2024

Year	Total HIV Tests	Total Persons Tested	Males Tested	Females Tested	Total New HIV(+)	New HIV(+) Males	New HIV(+) Females	Overall Rate per 1,000 Tested	Male Rate per 1,000 Tested	Female Rate per 1,000 Tested	HIV(+) Still in Military Service in 2024
2019	28,198	24,833	23,931	902	3	3	0	0.12	0.13	0.00	1
2020	19,371	17,874	17,141	733	2	2	0	0.11	0.12	0.00	1
2021	26,095	22,700	21,841	859	5	5	0	0.22	0.23	0.00	1
2022	19,963	17,747	17,024	723	3	3	0	0.17	0.18	0.00	3
2023	21,577	19,193	18,309	884	3	3	0	0.16	0.16	0.00	3
2024^a^	10,485	10,078	9,588	490	5	5	0	0.50	0.52	0.00	5
Total	125,689	112,425	107,834	4,591	21	21	0	0.19	0.19	0.00	14

Abbreviation: HIV, human immunodeficiency virus; +, positive.

^a^Through Jun. 30, 2024.

**Table 11:** New Diagnoses of HIV Infections, by Sex, Active Component, U.S. Coast Guard, January 2019–June 2024

Year	Total HIV Tests	Total Persons Tested	Males Tested	Females Tested	Total New HIV(+)	New HIV(+) Males	New HIV(+) Females	Overall Rate per 1,000 Tested	Male Rate per 1,000 Tested	Female Rate per 1,000 Tested	HIV(+) Still in Military Service in 2024
2019	21,219	20,342	17,192	3,150	2	2	0	0.10	0.12	0.00	0
2020	17,269	16,748	14,134	2,614	1	1	0	0.06	0.07	0.00	1
2021	20,464	19,801	16,633	3,168	1	1	0	0.05	0.06	0.00	1
2022	19,577	18,933	15,932	3,001	0	0	0	0.00	0.00	0.00	0
2023	19,280	18,488	15,477	3,011	0	0	0	0.00	0.00	0.00	0
2024^a^	9,395	9,221	7,685	1,536	1	1	0	0.11	0.13	0.00	1
Total	107,204	103,533	87,053	16,480	5	5	0	0.05	0.06	0.00	3

Abbreviation: HIV, human immunodeficiency virus; +, positive.

^a^Through Jun. 30, 2024.

**Table 12:** New Diagnoses of HIV Infections, by Sex, U.S. Coast Guard Reserve, January 2019–June 2024

Year	Total HIV Tests	Total Persons Tested	Males Tested	Females Tested	Total New HIV(+)	New HIV(+) Males	New HIV(+) Females	Overall Rate per 1,000 Tested	Male Rate per 1,000 Tested	Female Rate per 1,000 Tested	HIV(+) Still in Military Service in 2024
2019	2,617	2,471	2,069	402	1	1	0	0.40	0.48	0.00	0
2020	2,846	2,756	2,284	472	1	1	0	0.36	0.44	0.00	1
2021	3,233	3,027	2,514	513	0	0	0	0.00	0.00	0.00	0
2022	2,918	2,826	2,333	493	0	0	0	0.00	0.00	0.00	0
2023	2,908	2,789	2,277	512	0	0	0	0.00	0.00	0.00	0
2024^a^	1,698	1,659	1,358	301	0	0	0	0.00	0.00	0.00	0
Total	16,220	15,528	12,835	2,693	2	2	0	0.13	0.16	0.00	1

Abbreviation: HIV, human immunodeficiency virus; +, positive.

^a^Through Jun. 30, 2024.
